# Measurement properties of the Dutch versions of QuickDASH and PRWHE in patients with complaints of hand, wrist, forearm and elbow

**DOI:** 10.3233/BMR-230225

**Published:** 2024-07-02

**Authors:** Redmar J. Berduszek, Michiel F. Reneman, Rienk Dekker, Corry K. van der Sluis

**Affiliations:** University Medical Center Groningen, Department of Rehabilitation Medicine, University of Groningen, Groningen, The Netherlands

**Keywords:** Hand, upper extremity, patient reported outcome measures, pain measurement, disability evaluation, psychometrics

## Abstract

**BACKGROUND:**

The shortened version of the Disabilities of the Arm, Shoulder and Hand (QuickDASH) and Patient Rated Wrist/Hand Evaluation (PRWHE) are commonly used questionnaires to assess patient-reported hand function. Information about the measurement properties of the Dutch versions is scarce.

**OBJECTIVE:**

To gain insight into the measurement properties of the Dutch language versions of the QuickDASH and the PRWHE in patients with (non)specific complaints of the hand, wrist, forearm and elbow.

**METHODS:**

Internal consistency, construct validity, test-retest reliability, responsiveness, and floor and ceiling effects were assessed according to COnsensus-based Standards for the selection of health Measurement INstruments (COSMIN) recommendations.

**RESULTS:**

Questionnaires were filled out by 132 patients. Internal consistency of QuickDASH (Cronbach’s α= 0.92) and PRWHE (Cronbach’s α= 0.97) was high. Predefined hypotheses for construct validity were not confirmed for 75% for both QuickDASH and PRWHE (accordance with 62% of predefined hypotheses for both questionnaires). Test-retest reliability of QuickDASH (ICC = 0.90) and PRWHE (ICC = 0.87) was good. Both QuickDASH (AUC = 0.84) and PRWHE (AUC = 0.80) showed good responsiveness. No floor or ceiling effects were present.

**CONCLUSIONS:**

Measurement properties of the Dutch language versions of the QuickDASH and the PRWHE, applied to patients with (non)specific complaints of the hand, wrist, forearm and elbow, were very similar. Test-retest reliability and responsiveness were good for both QuickDASH and PRWHE. Construct validity could not be demonstrated sufficiently.

## 1. Introduction

Complaints of the arm, neck and shoulder (CANS) occur frequently in the Dutch population, with a point prevalence of over 25% and over half of the population reporting an episode of chronic complaints at least once during a 15-year course [1, 2]. Both pain and disability are considered important components of the assessment of hand problems [3]. Patient reported outcome measures (PROMs) are available to measure arm and hand function. Among the most frequently used region-specific questionnaires are the shortened version of the Disabilities of the Arm, Shoulder and Hand (QuickDASH) and Patient Rated Wrist/Hand Evaluation (PRWHE) [4, 5]. Both are brief self-report questionnaires, each taking less than 5 minutes to complete [4]. Both questionnaires, which are available in many languages including Dutch, have been considered essential tools to assess the outcome domain ‘patient-reported hand function/activities of daily living’ in patients with hand or wrist conditions [6].

Several measurement properties of QuickDASH and PRWHE have been studied widely, especially validity and reliability, while information on other measurement properties such as responsiveness is scarcer [7, 8]. Most studies have been performed in samples consisting of patients suffering from traumatic hand injury or other disorders requiring surgical intervention, but much less in patients with nontraumatic musculoskeletal complaints [7, 9]. Furthermore, the methodological quality of studies where measurement properties were assessed varies and is often low according to quality criteria for measurement properties [7, 8, 10, 11]. Measurement properties of the Dutch language versions of QuickDASH and PRWHE have been studied less extensively in general [12, 13]. To use and correctly interpret the results of these PROMs in a predominantly nontraumatic rehabilitation population, a better understanding of their measurement properties in such population is important [14].

Therefore, the aim of this study was to gain insight into the measurement properties (internal consistency, construct validity, test-retest reliability, responsiveness (including the minimal important change (MIC) value) and floor or ceiling effects) of the Dutch language versions of QuickDASH and PRWHE in patients with nontraumatic musculoskeletal complaints of the hand, wrist, forearm and elbow.

## 2. Methods

### 2.1 Study design

The design of this prospective observational study was based on the recommendations of the COnsensus-based Standards for the selection of health Measurement INstruments (COSMIN) initiative [15, 16]. This study was approved by the Medical Ethical Committee of the University Medical Center Groningen (METc 2015/115) and has been registered with the Dutch Trial Register (NL5657). All participants gave written informed consent.

### 2.2 Study sample

Participants were recruited between November 2015 and March 2020. Participants were originally selected from patients visiting the outpatient clinic of the department of rehabilitation medicine of one university hospital and, to expedite inclusion, since January 2017 also from two primary care hand therapy clinics located in the same region as the university hospital. Participants were eligible if they were 18 years or older and had musculoskeletal complaints of their hand, wrist, forearm and/or elbow. These complaints were classified as specific or nonspecific CANS, according to the CANS model [17]. CANS is defined as musculoskeletal complaints of arm, neck and/or shoulder not caused by acute trauma or by any systemic disease. While CANS covers disorders located as proximally as neck and shoulder, participants in this study were affected by more distally located complaints (elbow and more distal) directly influencing hand function. This also included lateral epicondylitis, which involves the muscles and tendons of the forearm that extend the wrist and fingers. Exclusion criteria were insufficient understanding of the Dutch language to fill out questionnaires, disorders excluded by the CANS model (e.g., osteoarthritis, rheumatoid arthritis) and the presence of concomitant medical conditions causing considerate disability, such as neurological disorders (e.g., stroke, traumatic peripheral nerve damage) or (partial) amputation of the hand. Participants were selected through convenience sampling.

Intended sample size was based on COSMIN recommendations: at least 50 subjects to assess construct validity, reliability, responsiveness and floor or ceiling effects, and at least 7 times the number of items of a questionnaire (with a minimum of 100) to assess internal consistency (in this case the questionnaire with the most items was the PRWHE (15 items), therefore 7 × 15 = 105) [15].

**Table 1:** Construct validity: predefined hypotheses and results

	Predefined hypothesis	Correlation with QuickDASH (ρ) (accepted yes/no)	Correlation with PRWHE (ρ) (accepted yes/no)
QuickDASH/PRWHE	> 0.75	0.87 (yes)	0.87 (yes)
PDI	0.26–0.75	0.88 (no)	0.87 (no)
NRS Pain	0.26–0.75	0.75 (yes)	0.84 (no)
RAND-36 physical functioning	0.26–0.75	-0.70 (yes)	-0.64 (yes)
RAND-36 social functioning	0.00-0.50	-0.61 (no)	-0.54 (no)
RAND-36 vitality	0.00-0.50	-0.59 (no)	-0.45 (yes)
RAND-36 mental health	0.00-0.25	-0.47 (no)	-0.38 (no)
WAS	0.26-0.50	-0.67 (no)	-0.63 (no)
Age	0.00–0.25	0.03 (yes)	0.03 (yes)
Hand grip strength^†^	0.26–0.75	-0.43 (yes)	-0.43 (yes)
	Predefined hypothesis	Group comparison (accepted yes/no)	Group comparison (accepted yes/no)
Score: lower if employed	Yes	Yes, p= 0.009 (yes)	Yes, p= 0.002 (yes)
Score: male = female	Yes	Yes, p= 0.53 (yes)	Yes, p= 0.94 (yes)
Score: higher if dominant side affected	No	No, p= 0.47 (yes)	No, p= 0.14 (yes)

QuickDASH: shortened version of the Disabilities of the Arm, Shoulder and Hand, PRHWE: Patient Rated Wrist/Hand Evaluation, PDI: Pain Disability Index, NRS Pain: Numeric Pain Rating Scale, RAND-36: RAND 36-item Health Survey, WAS: Work Ability Score. *0.00–0.25: weak; 0.26–0.50: moderate; 0.51–0.75: strong; > 0.75: very strong [32]. †Grip strength of the affected hand, or dominant hand in case of bilateral involvement.

### 2.3 Procedure

**Figure 1.**
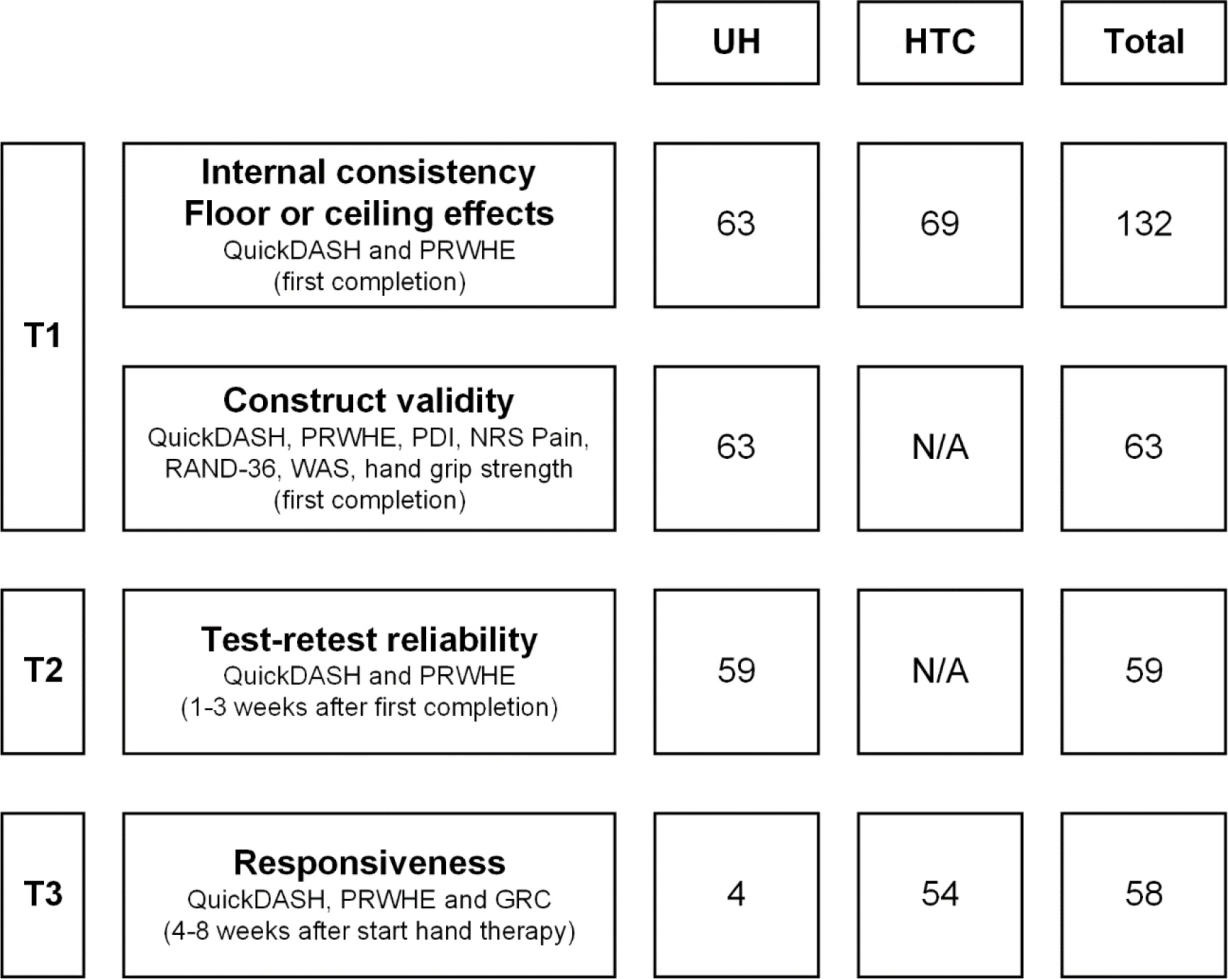
Flowchart of study procedure and number of participants. Overview of the three study measurement moments (T1, T2 and T3), the measures taken per moment (depending on the measurement properties studied) and the number of participants for each measurement property and location. UH: university hospital, HTC: primary care hand therapy clinics, QuickDASH: shortened version of the Disabilities of the Arm, Shoulder and Hand, PRHWE: Patient Rated Wrist/Hand Evaluation, PDI: Pain Disability Index, NRS Pain: Numeric Pain Rating Scale, RAND-36: RAND 36-item Health Survey, WAS: Work Ability Score, GRC: global rating of change, N/A: not applicable.

Participants filled out questionnaires two or three times (at T1, T2 and/or T3), depending on inclusion location and whether they were treated by a certified hand therapist at the institution where they were included (Fig. 1). Questionnaires were paper-based and handed out during a consultation (T1 at hand therapy clinics) or distributed by post (T1 and T2 at the university hospital, T3 at the university hospital and hand therapy clinics). In any case, participants could fill out the questionnaires at a self-selected moment and return them by post. The interval between T1 and T2 was 1–3 weeks, which was supposed to be long enough to prevent recall and allow administration of questionnaires by post, yet short enough to assume no clinical change occurred [16]. University hospital participants with a site visit within 1 week of T1 (n= 45) also performed a hand grip strength measurement, which was used to assess construct validity.

### 2.4 Measurements

All participants filled out general demographic information regarding marital status, level of education, current work situation and handedness. Diagnosis was recorded from the medical record.

#### 2.4.1 Primary measures

The QuickDASH is a shortened version of the Disabilities of the Arm, Shoulder and Hand (DASH) questionnaire, which was developed to measure physical function and symptoms in persons with musculoskeletal disorders of the upper limb [18, 19]. It consists of 11 items regarding function (7 items) and pain (4 items). The total score ranges from 0–100, where a higher score indicates more pain and disability. Internal consistency, construct validity, reliability and responsiveness have been rated to be adequate (mainly in patients with neck or shoulder disorders, patients with fractures or other injuries, or surgically treated patients) [8, 10]. 

The PRWHE is a questionnaire which was developed to assess pain and disability of the wrist and hand [20, 21, 22]. It consists of 15 items divided over two subscales regarding pain (5 items) and function (10 items) of wrist and hand. Both pain and function contribute equally to the total score, which ranges from 0–100. A higher score indicates more pain and disability. Its measurement properties have been assessed in diverse countries and populations (mainly in patients with fractures or other injuries), generally demonstrating very good internal consistency, construct validity and reliability [7].

#### 2.4.2 Secondary measures

**Table 2:** Participant characteristics

	Total (n= 132)	UH (n= 63)	HTC (n= 69)
Sex (male) (n (%))	49 (37)	23 (37)	26 (38)
Age (years) (mean (SD))	47.4 (16.7)	45.1 (15.1)	49.5 (17.9)
Diagnosis (n (%))			
Specific CANS	73 (55)	25 (40)	48 (70)
Lateral epicondylitis	20 (28)	7 (28)	13 (27)
De Quervain’s disease	19 (26)	7 (28)	12 (25)
Trigger finger	17 (23)	7 (28)	10 (21)
Carpal tunnel syndrome	9 (12)	0 (0)	9 (19)
Dupuytren disease	8 (11)	4 (16)	4 (8)
Nonspecific CANS	59 (45)	38 (60)	21 (30)
Handedness (n (%))			
Right-handedness	108 (82)	51 (81)	57 (83)
Left-handedness	14 (11)	9 (14)	5 (7)
Mixed or ambidexterity	10 (7)	3 (5)	7 (10)
Affected side (n (%))			
Unilateral	79 (60)	35 (56)	44 (64)
Bilateral	53 (40)	28 (44)	25 (36)
Dominant hand affected (yes) (n (%))	107 (82)	51 (81)	56 (81)
QuickDASH (0–100, median (IQR))	31.8 (29.0)	31.8 (29.6)	34.1 (30.7)
PRWHE (0–100, median (IQR))	46.8 (43.0)	41.5 (42.0)	50.0 (42.5)
Employed (yes) (n (%))	92 (70)	48 (76)	44 (64)
WAS (0–10, mean (SD))	-	5.7 (2.8)	-
NRS Pain (0–10, mean (SD))	-	4.5 (2.3)	-

UH: university hospital. HTC: primary care hand therapy clinics. CANS: complaints of the arm, neck and shoulder. QuickDASH: shortened version of the Disabilities of the Arm, Shoulder and Hand. PRWHE: Patient Rated Wrist/Hand Evaluation. WAS: Work Ability Score. NRS Pain: Numeric Pain Rating Scale.

Secondary measures were collected depending on location and the time the questionnaires were filled out (Fig. 1), to assess either construct validity or responsiveness. The Pain Disability Index (PDI) is a generic instrument for measuring disability related to pain. It consists of 7 items concerning self-reported disability due to pain in different situations such as work, leisure time, activities of daily living, and sports. The total score ranges from 0–70. A higher score reflects a greater disability due to pain. It has been proven valid and reliable in patients with different types of musculoskeletal pain [23].

The Numeric Pain Rating Scale (NRS Pain) is a valid and reliable, unidimensional scale to assess pain intensity [24]. It consists of a single item asking about pain intensity during the past week. It is scored on an 11-point Likert scale ranging from 0 (no pain) to 10 (worst pain imaginable).

The RAND 36-item Health Survey (RAND-36) is a questionnaire about physical, mental and social health and is used worldwide to measure health-related quality of life, which has been shown to be reliable and valid [25]. The RAND-36 is a license free version of the SF-36 and includes the same items [26]. It consists of eight subscales measuring either physical or mental health. While the complete RAND-36 was filled out, only the subscales physical functioning, social functioning, vitality, and mental health were analyzed in this study for the purpose of construct validity assessment. Subscale scores are calculated using an algorithm, the output being a score between 0 and 100. Higher scores indicate a better health status.

The Work Ability Score (WAS) is a single-item questionnaire asking about the current work ability compared to the lifetime best work ability, ranging from 0 (completely unable to work) to 10 (lifetime best work ability). It has been shown to be a valid, reliable, and responsive instrument to assess current work ability [27].

Hand grip strength was measured using a Jamar dynamometer, the patient sitting with the elbow flexed at 90 degrees and the forearm and wrist in a neutral position. Both hands were assessed three times each in alternating order and the mean for each hand was calculated [28].

A question about the global rating of change (GRC) was used as an external criterion to assess clinically meaningful change, in order to assess responsiveness [29]. Participants who were treated by a certified hand therapist (at their inclusion location, either the university hospital or one of the two hand therapy clinics) were asked to rate the perceived change in complaints of their hand, wrist or forearm since the start of hand therapy on a 7-point Likert scale, ranging from 1 (much better) to 7 (much worse). In general, hand therapy treatments included exercises, ergonomic advice and relative rest (e.g., splinting).

### 2.5 Data analyses

Statistical analyses were performed using IBM SPSS Statistics 28. Descriptive statistics were used to describe patient characteristics. Parametric or nonparametric statistics were used where appropriate. Statistical significance was set at p< 0.05.

#### 2.5.1 Internal consistency

Cronbach’s α was calculated for each (sub)scale, a value between 0.70 and 0.95 was considered adequate [15].

#### 2.5.2 Construct validity

Construct validity of QuickDASH and PRWHE was evaluated through 13 predefined hypotheses (Table 1). Because of their equivalent construct, these hypotheses were the same for both QuickDASH and PRWHE.

The hypotheses were based on a theoretical assessment of the concepts being measured. Both QuickDASH and PRWHE assess pain of the arm/hand, the ability to use the hand and to perform daily activities. As such, a very strong relationship between these two questionnaires was expected. Also, because of their similar construct, the assumed strength of the correlation with other variables was identical for both QuickDASH and PRWHE. The PDI measures the impact of pain on the ability of a person to participate in essential life activities but does not focus specifically on the upper extremities. Similarly, the RAND-36 subscale physical functioning is composed of items assessing the influence of health problems on different physical activities, some involving the upper extremity. Therefore, a moderate to strong relationship between QuickDASH/PRWHE and PDI/RAND-36 subscale physical functioning was expected. A similar relationship was expected between QuickDASH/PRWHE and WAS, as it is perceivable that upper extremity pain and disabilities have some effect on work ability. Because pain contributes partially to the total scores of QuickDASH/PRHWE, a moderate to strong correlation with NRS Pain was expected. The correlation between QuickDASH/PRWHE and RAND-36 subscales social functioning, vitality and mental health was expected to be weak to moderate, because these subscales test constructs not directly related to upper extremity function. A moderate to strong correlation between QuickDASH/PRWHE and hand grip strength was expected, since hand grip strength might be affected by the disorder or associated pain. Age and sex influence QuickDASH/PRWHE scores only slightly, therefore a weak correlation with age and no differences between males and females were expected [30, 31]. It was assumed that better hand function was reported by those who were working, therefore lower QuickDASH/PRWHE scores were expected in participants who were employed opposed to unemployed. Because use of the dominant hand is not assumed for the activities listed in QuickDASH/PRWHE, no difference in QuickDASH/PRWHE scores was expected between participants of which the dominant side was affected or not.

Spearman’s rank correlation coefficients (r) were calculated to assess associations with other measurements. Correlation coefficients were interpreted as follows: 0.00–0.25 weak, 0.26–0.50 moderate, 0.51–0.75 strong, above 0.75 very strong [32]. Known-group differences were assessed using the Mann-Whitney U test. Construct validity was deemed good when at least 75% of the results were in accordance with the predefined hypotheses [15].

#### 2.5.3 Test-retest reliability

An intraclass correlation coefficient (ICC) for absolute agreement (two-way mixed effects model) was calculated, an ICC ⩾ 0.70 was deemed good [15]. The 95% Limits of Agreement (LoA) were presented using a Bland-Altman plot. LoA are defined as the mean difference between repeated measurements ± 1.96 SD of the difference [33].

#### 2.5.4 Responsiveness

The GRC was used as an external criterion (anchor-based method) [29]. A score of 1 or 2 ((much) improved) was considered as an improvement, a score of 3 (slightly improved), 4 (the same) or 5 (slightly worse) was considered unchanged, and a score of 6 or 7 ((much) worse) was considered as a deterioration of complaints. The area under the receiver operating characteristics (ROC) curve (AUC) was calculated to assess discrimination between participants whose complaints had improved versus remained unchanged [34]. An AUC of at least 0.70 was considered adequate to distinguish between patients who have improved versus remained unchanged [15]. The MIC (the smallest change in the score that patients perceive as important) was determined by the ROC cut-off point associated with optimal sensitivity and specificity, using the sum of squares approach [35]. This approach determines the ROC cut-off point by finding the smallest sum of squares of 1-sensitivity and 1-specificity, assuming sensitivity and specificity are valued equally. The standard error of measurement (SEM) was calculated by the square root of the error variance of an ANOVA analysis including systematic differences (SEM_agreement_). The smallest detectable change (SDC, the smallest change that can be detected beyond measurement error) was calculated using the formula SDC = 1.96 ×√2 × SEM. The SDC should be smaller than the MIC, to distinguish between clinically meaningful change and measurement error [15]. 

#### 2.5.5 Floor or ceiling effects

Floor or ceiling effects were considered to be present if more than 15% of participants achieved the lowest or highest possible score [15].

## 3. Results

The QuickDASH and PRWHE were filled out by 132 patients, 63 at the university hospital and 69 at the hand therapy clinics (Table 2). The number of participants included in the analysis of each measurement property ranged from 58 to 132 (Fig. 1). Specific CANS were relatively more prevalent in patients included from the hand therapy clinics compared to those included from the university hospital ($X^{2}$ (df = 1, n= 132) =11.90, p= 0.001), where nonspecific CANS were more prevalent. Other characteristics did not differ significantly between university hospital and hand therapy clinic populations.

### 3.1 Internal consistency

For QuickDASH, Cronbach’s α was 0.92. For PRWHE, Cronbach’s α of the complete questionnaire (15 items) was 0.97, for the pain subscale (5 items) and for the disability subscale (10 items) it was 0.93 and 0.96, respectively.

### 3.2 Construct validity

Accordance with predefined hypotheses was observed in 8 of 13 (62%) hypotheses tested for both QuickDASH and PRWHE (Table 1), meaning that construct validity of both questionnaires could not be demonstrated.

**Figure 2.**
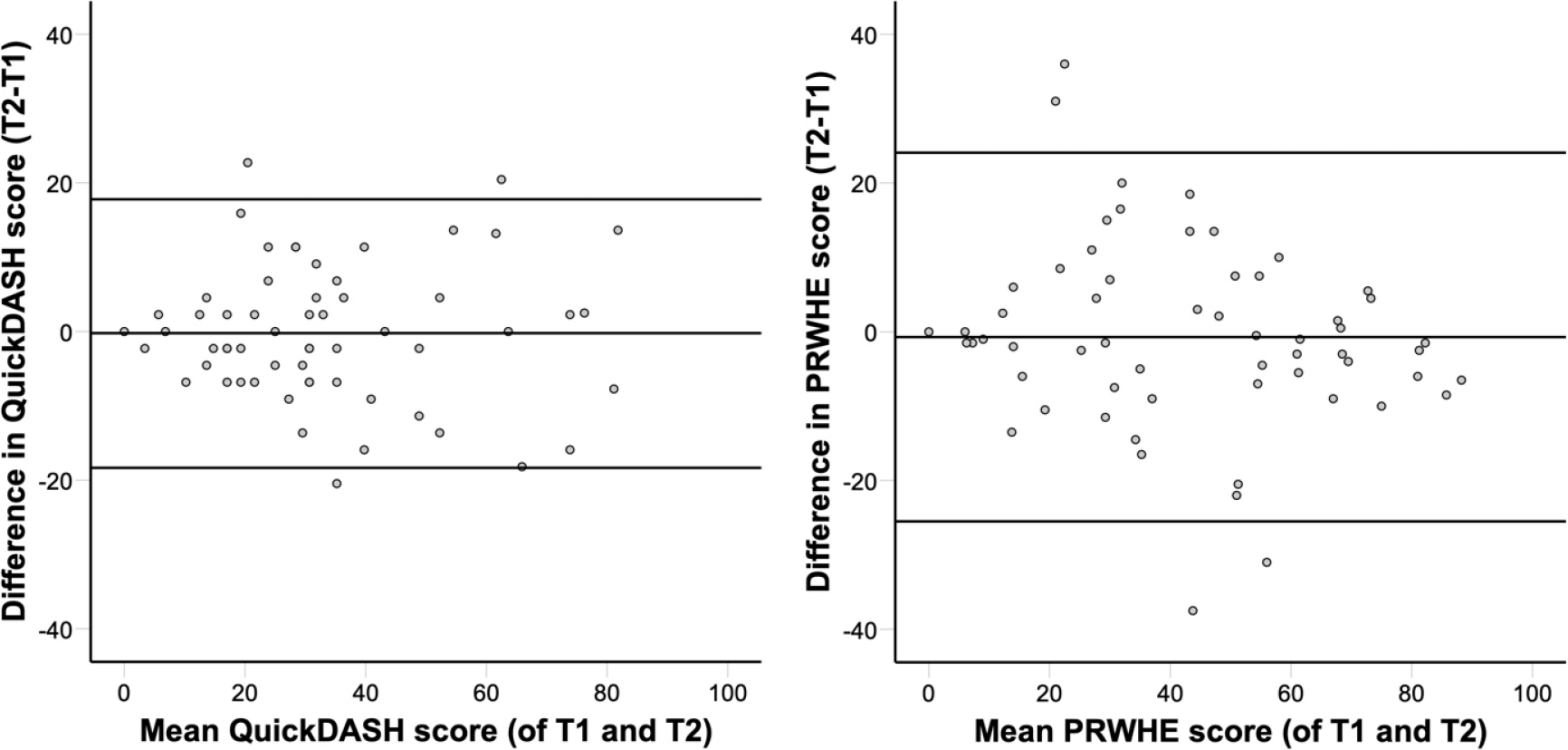
Bland-Altman plots for QuickDASH and PRWHE. Bland-Altman plots of differences between scores at the first measurement moment (T1) and second measurement moment (T2, 1–3 weeks after T1) versus the mean of these two measurements. For QuickDASH (left panel), the mean difference between T1 and T2 was -0.21 with LoA of -18.34 (lower) and 17.92 (upper). For PRWHE (right panel), the mean difference between T1 and T2 was -0.71 with LoA of -25.50 (lower) and 24.08 (upper). LoA: 95% Limits of Agreement, QuickDASH: shortened version of the Disabilities of the Arm, Shoulder and Hand, PRHWE: Patient Rated Wrist/Hand Evaluation.

### 3.3 Test-retest reliability

For QuickDASH, ICC was 0.90 (95% CI: 0.84–0.94). The mean difference between test and retest was -0.21 with LoA of -18.34 (lower) and 17.92 (upper) (Fig. 2). For PRWHE, ICC was 0.87 (95% CI: 0.78–0.92). The mean difference between test and retest was -0.71 with LoA of -25.50 (lower) and 24.08 (upper) (Fig. 2).

### 3.4 Responsiveness

Of the 58 participants who received hand therapy treatment, 21 (36%) indicated that their complaints had improved since the start of this treatment (GRC score 1 or 2), while 35 (60%) indicated that their complaints were unchanged (GRC score 3, 4 or 5). Participants whose complaints had improved after hand therapy had a greater reduction of both QuickDASH (mean difference 21.3, 95% CI: 12.2-30.4, p< 0.001) and PRWHE (mean difference 18.4, 95% CI: 9.3–27.6, p< 0.001) scores compared to participants whose complaints were unchanged.

**Figure 3.**
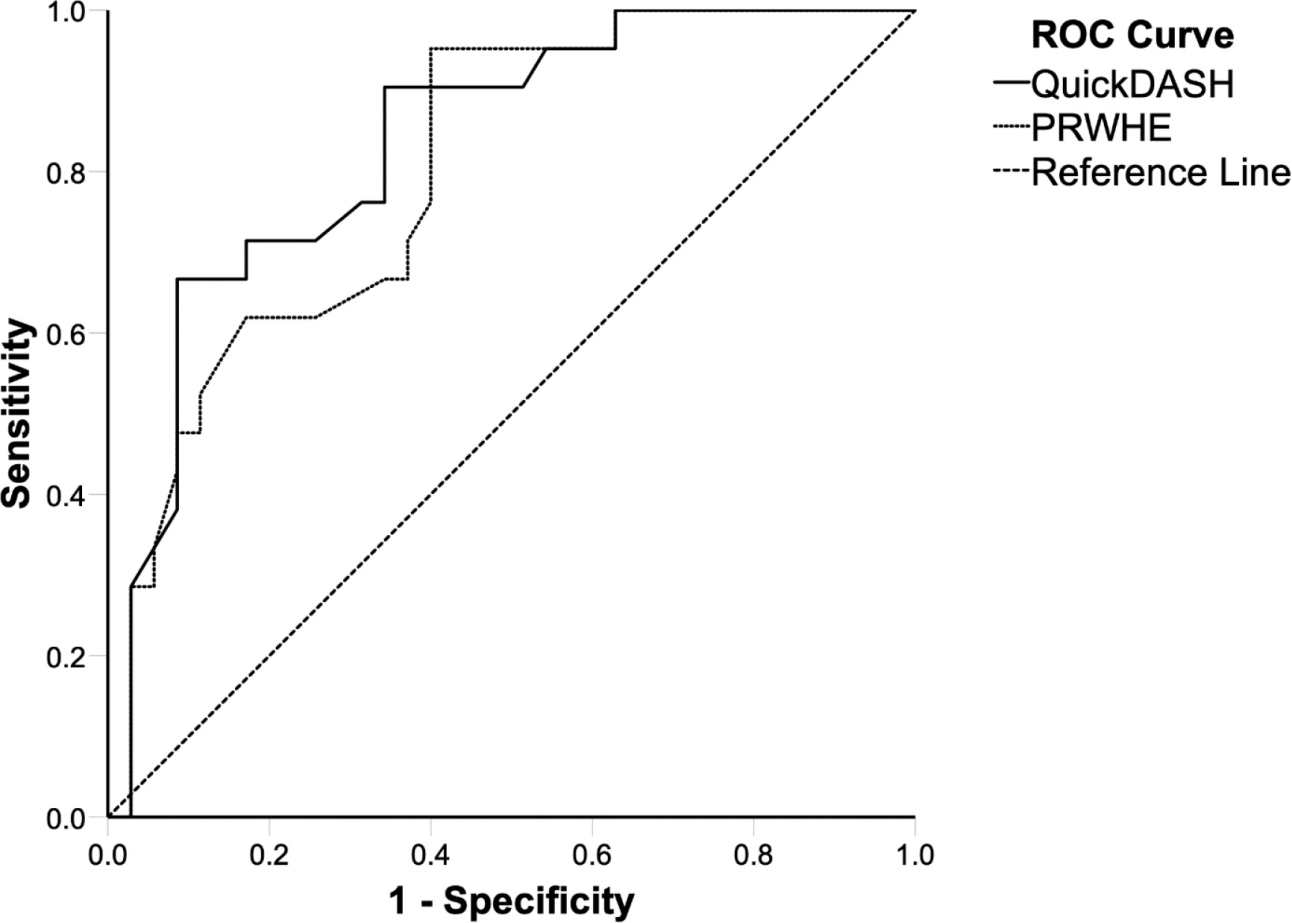
ROC curves for QuickDASH (solid line) and PRWHE (dotted line) represented in comparison to a reference line (dashed line). The AUC was calculated to assess discrimination between participants whose complaints had improved versus remained unchanged. The AUC was 0.84 (95% CI: 0.73–0.94) for QuickDASH and 0.80 (95% CI: 0.69–0.92) for PRWHE. ROC: receiver operating characteristics, AUC: area under the ROC curve, QuickDASH: shortened version of the Disabilities of the Arm, Shoulder and Hand, PRHWE: Patient Rated Wrist/Hand Evaluation.

The AUC was 0.84 (95% CI: 0.73–0.94) for QuickDASH and 0.80 (95% CI: 0.69–0.92) for PRWHE (Fig. 3). For QuickDASH, the ROC cut-off and MIC was 15.9 points (sensitivity 0.71, specificity 0.83), the SEM was 1.85 and the SDC was 5.13. For PRWHE, the ROC cut-off and MIC was 10.3 points (sensitivity 0.95, specificity 0.60), the SEM was 2.15 and the SDC was 5.96. 

### 3.5 Floor or ceiling effects

For both QuickDASH and PRWHE, only one of 132 participants had the lowest possible score (less than 1%). None of the participants had the highest possible score on either of these questionnaires. This indicates that no floor or ceiling effects were present.

## 4. Discussion

This study assessed multiple measurement properties of QuickDASH and PRWHE in a Dutch rehabilitation population suffering from complaints of hand, wrist, forearm and/or elbow, classified as specific or nonspecific CANS. Outcomes were compared to COSMIN quality criteria for measurement properties [15]. The quality of measurement properties of QuickDASH and PRWHE were similar in this study sample. Most measurement properties were sufficient: internal consistency, test-retest reliability, responsiveness and floor and ceiling effects for both QuickDASH and PRWHE. Construct validity was insufficiently demonstrated for both QuickDASH and PRHWE, because less than 75% of results were in accordance with predefined hypotheses.

Internal consistency of QuickDASH and PRWHE in previous studies was invariably high and similar to our findings [7, 8, 9, 11, 36]. The very high Cronbach’s α of PRWHE disability subscale is indicative of item redundancy. Using factor analysis, multiple studies have demonstrated that PRWHE actually consists of three (pain, specific activities, usual activities) instead of two subscales (pain and function) [37, 38, 39].

Construct validity of QuickDASH and PRWHE in this study was insufficient, because less than 75% of observed correlations with other parameters were in accordance with predefined hypotheses. The correlations between QuickDASH/PRWHE and PDI, WAS and most RAND-36 subscales were stronger than hypothesized. While the strength of observed correlations matched with expected correlations for only 62% of the predefined hypotheses, the order of the observed correlation coefficients did correspond with those of the hypotheses. So, the RAND-36 subscale physical functioning was correlated more strongly with QuickDASH/PRWHE than the RAND-36 subscales social functioning and vitality. Also, as expected, the weakest correlation was observed for the RAND-36 subscale mental health. In two studies assessing the measurement properties of QuickDASH (Chinese version) and PRWHE (Turkish version) in patients with diverse upper extremity disorders, correlations with the subscales physical functioning, social functioning, vitality and mental health of 36-Item Short-Form Health Survey (SF-36, which resembles RAND-36 strongly) were assessed. The same order of correlation coefficients was described as in this study, yet the strength of these correlations was much weaker and within the ranges hypothesized in this study [37, 40]. In a study assessing the measurement properties of the QuickDASH in patients with acute elbow trauma, a strong correlation was found between QuickDASH and SF-36 subscale physical functioning (similar to the correlation between QuickDASH and RAND-36 subscale physical functioning in this study), but a much weaker correlation between QuickDASH and SF-36 mental component scale (consisting of four SF-36 domains, amongst which social functioning, vitality and mental health) than between QuickDASH and RAND-36 subscales social functioning, vitality and mental health in this study [13]. While we carefully considered the synthesis of the predefined hypotheses, we argue that we could have been less strict in describing the precise strength of expected correlation coefficients. Even though alternative explanations might introduce bias, we were more confident about the relative than the absolute magnitude of the correlations. Therefore, we feel that both QuickDASH and PRWHE might be more valid than demonstrated in this study. In any case, the results provide more insight into the construct of both questionnaires in a sample of patients with nontraumatic musculoskeletal complaints of the hand, wrist, forearm and elbow.

Test-retest reliability of both QuickDASH andPRWHE was very good, which matches previous literature reporting similar reliability almost without exception [7, 8, 9, 11].

Responsiveness of both QuickDASH and PRWHE was good, with an AUC of over 0.70 and SDC smaller than MIC for both QuickDASH and PRWHE, indicating that clinically important change can be distinguished from measurement error. QuickDASH had a MIC of 16 points and PRHWE had a MIC of 10 points. Previously reported MIC differed amongst others between diagnoses and treatment type (generally lower for nonsurgical treatment compared to surgical treatment), but are similar to our findings for both QuickDASH (range 14–18) and PRWHE (range 13–14) in similar samples [41, 42, 43, 44]. The cut-off point to determine MIC may be chosen differently, depending on the preferred balance between sensitivity and specificity (see Appendix for cut-off values and associated sensitivity and specificity) [34].

### 4.1 Clinical implications and suggestions for further research

While construct validity and floor and ceiling effect results were on par between QuickDASH and PRWHE, internal consistency, test-retest reliability and responsiveness of QuickDASH seemed slightly favorable over those of PRWHE. Furthermore, QuickDASH consists of fewer items and can be used in a wider population experiencing problems anywhere in the upper extremity, while PRWHE focuses on wrist and hand problems. Therefore, we consider that use of the QuickDASH may be preferred over the PRWHE. Due to the instructions of the PRWHE (pain in hand/wrist) its use in patients with pain in the forearm or elbow may be limited (even when this pain is directly related to hand function). Small changes to these instructions might be considered to broaden its application [45]. Expansion of these insights may support decisions regarding the use of these questionnaires in clinical practice and contribute to the further development of a viable methodology for use in research on patients with upper limb disability [46].

Suggestions for further research include further validation of the QuickDASH and PRWHE as well as the assessment of their measurement properties in different, but more homogenous populations (e.g., test-retest reliability should be evaluated additionally in a primary care population and responsiveness should be evaluated additionally in a tertiary care population). Also, because of possible item redundancy, further shortening or division in subscales of these questionnaires deserves attention.

### 4.2 Limitations

Despite adherence to COSMIN guidelines, COSMIN recommended sample sizes have been increased in recent design checklists and the sample size used in this study is currently considered as adequate instead of very good [47]. The sample size did not allow for a further division into groups of diagnoses. Stability on the construct measured during the interval for test-retest reliability was assumed but not assessed on an individual level. While all participants had similar disorders, there was a difference in distribution of specific versus nonspecific CANS between university hospital and primary care hand therapy clinic populations. Not all upper extremity regions were represented in the population studied (e.g., no shoulder disorders). Also, even though participants originated from the same geographical area, the fact that some of them were seen in primary care and others in tertiary care might limit generalizability.

## 5. Conclusion

Measurement properties of the Dutch language versions of QuickDASH and PRWHE, applied to patients with (non)specific complaints of the hand, wrist, forearm and elbow, were very similar. Internal consistency was slightly better for QuickDASH than PRWHE. Test-retest reliability and responsiveness were good for both QuickDASH and PRWHE. Construct validity could not be demonstrated sufficiently. No floor or ceiling effects were present.

## Author contributions

Study conception and design: all authors. Data collection and analysis: RB. Drafting of the manuscript: RB. Critical revision of the manuscript: all authors. All authors read and approved the final manuscript.

## Data availability statement

Data are available from the corresponding author upon reasonable request.

## Ethical approval

The study has been performed in accordance with the Declaration of Helsinki and amendments and was approved by the Medical Ethical Committee of the University Medical Center Groningen (2015/115).

## Funding

The authors report no funding.

## Informed consent

All participants gave written informed consent.
